# Titanium nanosheet as robust and biosafe drug carrier for combined photochemo cancer therapy

**DOI:** 10.1186/s12951-022-01374-0

**Published:** 2022-03-24

**Authors:** Xiaoli Yuan, Ying Zhu, Shasha Li, Yiqun Wu, Zhongshi Wang, Rui Gao, Shiyao Luo, Juan Shen, Jun Wu, Liang Ge

**Affiliations:** 1 State Key Laboratory of Natural Medicines, China Pharmaceutical University, No. 24 Tongjia Xiang, Nanjing, 210009 China; 2 School of Biomedical Engineering, Sun Yat-Sen University, Guangzhou, 510006 China; 3 School of Pharmacy, Xinjiang Medical University, Xinjiang, 830000 China

**Keywords:** Titanium nanosheet, Polydopamine modification, Chemo-photothermal therapy, Drug delivery

## Abstract

Two-dimensional (2D) Titanium nanosheets (Ti NSs) have shown many excellent properties, such as nontoxicity, satisfactory photothermal conversion efficacy, etc. However, the biomedical applications of Ti NSs have not been intensively investigated. Herein, we synthesized a multifunctional Ti NS drug delivery system modified with polydopamine/polyethylene glycol (Ti@PDA-PEG) and applied simultaneously for photothermal therapy and chemotherapy. Doxorubicin (DOX) was utilized as a model drug. Ti@PDA-PEG NS shows an ultrahigh antitumor drug DOX loading (Ti@PDA-PEG-DOX). The prepared Ti@PDA-PEG-DOX NS as robust drug delivery system demonstrates great stability and excellent multi-response drug-release capabilities, including pH-responsive and near-infrared -responsive behavior and obviously high photothermal efficiency. Both in vitro and in vivo experimental results have shown high biosafety and outstanding antitumor effects. Therefore, this work exhibits the enormous potential of a multifunctional platform in the treatment of tumors and may stimulate interest in the exploration of other new 2D nanomaterials for biomedical applications.

**Graphical Abstract:**

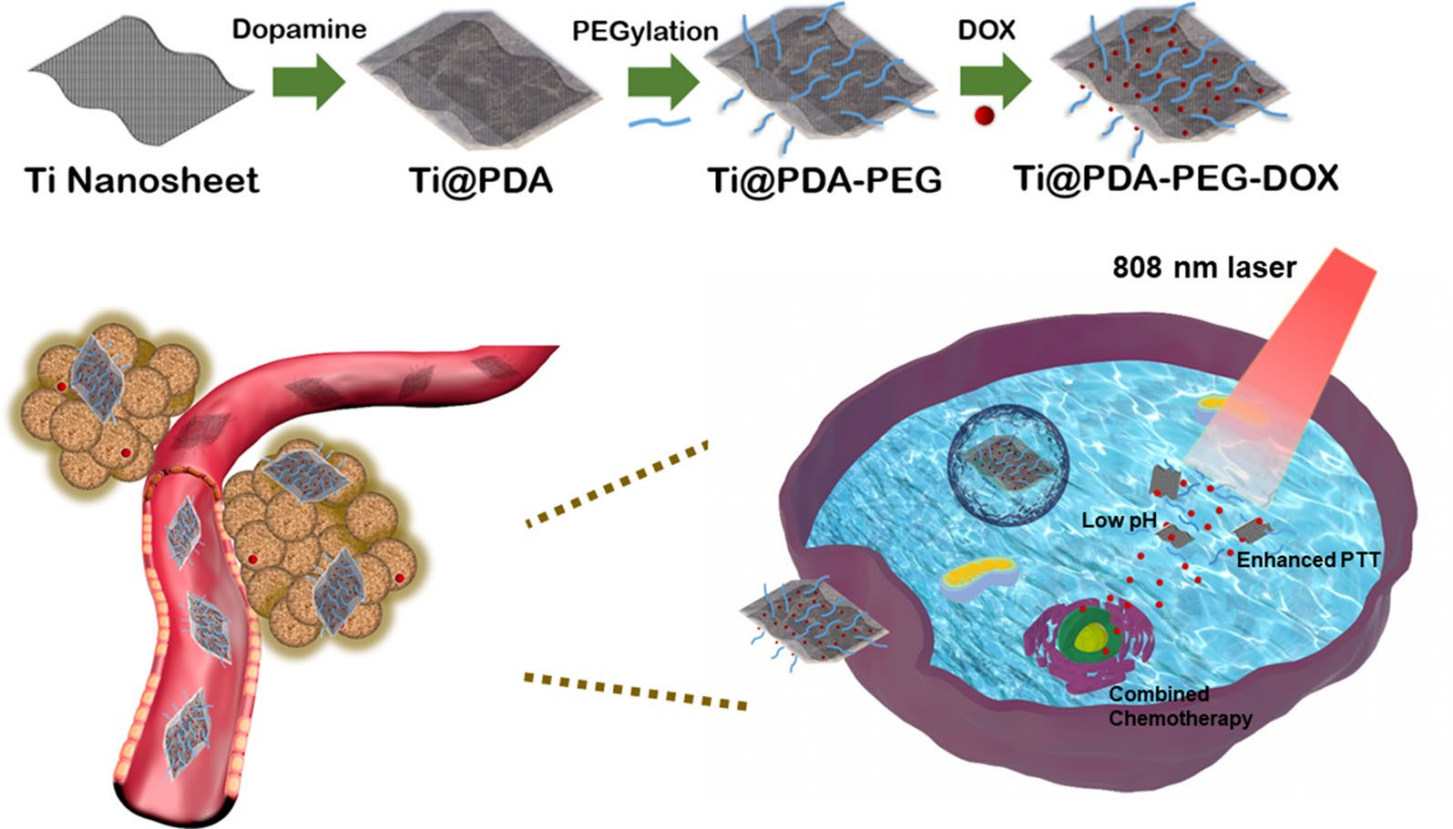

**Supplementary Information:**

The online version contains supplementary material available at 10.1186/s12951-022-01374-0.

## Introduction

Photothermal therapy (PTT) is a typical antitumor technology. Photoenergy is converted into heat by photothermal agents (PTAs) under near-infrared (NIR) laser irradiation at tumor sites, which can destroy tumor tissues without harming healthy tissues by providing a spatiotemporally controllable thermal effect within the light-irradiated area [1]. However, single-photothermal therapy has difficulty eliminating tumor cells due to light scattering and absorption in tumor sites [2–4]. Therefore, PTT in combination with chemotherapy has attracted widespread attention in tumor treatment. According to reports [5, 6], combination therapy was better than individual therapeutic models for antitumor therapy, which could improve tumor treatment efficacy, elevate long-term prognosis and reduce drug side effects.

Recently, two-dimensional (2D) nanomaterials have been considered promising PTAs in the field of PTT and other antitumor therapy combined with PTT. In 2004, graphene was successfully prepared [7], which was a milestone among 2D nanomaterials. Then, an increasing number of 2D nanomaterials have been explored [8], such as graphene derivatives, MXenes [9], BP nanosheets (NSs) [10], transitional metal dihalcogenides [11, 12], and Pd NSs [13]. Compared to other nanomaterials, 2D nanomaterials have many advantages including the number of layers of 2D nanomaterials being changed to adjust their optical properties [14] and their large surface area loading other molecules for combined therapy [15]. Titanium (Ti) and its alloys, such as Ti_3_C_2_ [16], TiS_2_, and TiN, have attracted extensive research interest in various fields, including physics, energy evolution and medical materials. In particular, Ti and its alloys have become one of the most attractive materials in the field of biomaterials for the replacement or repair of artificial joints, dental implants, interventional cardiovascular stents, etc. Ti NS, as a new and economic 2D nanomaterial, has shown satisfactory photothermal conversion efficacy and good biosafety. However, there are few reports about the biomedical applications of 2D Ti NSs, especially in the field of drug delivery systems.

According to a previous report [17], Ti NSs displayed strong NIR light absorbance and outstanding photothermal conversion capability, and the photothermal conversion efficiency (PTCE) value was calculated to be as high as 61.5%. The PTCE value of Ti NSs was significantly larger than that of other important photothermal agents, such as Au nanoparticles (21%) [18], MoS_2_ (24.4%) [19], and antimonene NSs (41.8%) [20]. However, the application of 2D Ti NSs in medical materials is largely limited, partially due to their instability and easy oxidation in vitro and in vivo. Herein, a polydopamine (PDA) layer was coated on the surface of Ti NS to improve its property [21]. Since 2007, Messersmith et al. [22] proposed a simple surface modification method based on dopamine. In recent years, PDA modification has been widely used in the biological field due to its high biocompatibility, excellent functional platform, biodegradation at low pH values and potential photothermal properties. On the whole, the PDA shell could not only improve Ti NS stability but also further serve as a platform for secondary modification. To improve the therapeutic effect and safety, polyethylene glycol (PEG) was introduced to increase the retention time of NSs in the blood circulation and enhance the drug delivery ability at the tumor site [23]. In addition, it is crucial to load effective antitumor drug on nanomaterials to strengthen antitumor function.

In this work, we designed and fabricated a multifunctional nanoplatform based on Ti NSs applied simultaneously for photochemo tumor therapy, which utilized Ti@PDA-PEG NSs as PTAs and doxorubicin (DOX) as the chemotherapeutic agent. The PDA film was coated on Ti NSs to stabilize the nanoplatform before use in vivo and further connected to mPEG-SH via Michael addition. Finally, DOX-loaded Ti@PDA-PEG NSs formed the entire drug delivery system (Ti@PDA-PEG-DOX). Under NIR laser irradiation, the prepared multifunctional nanoplatform for PTT combined with chemotherapy showed significant tumor inhibition in vitro and in vivo. Based on these studies, the Ti@PDA-PEG-DOX NS drug delivery platform could be promising in antitumor treatment, which also further boosted the research of other novel 2D nanomaterials in the field of biomedicine.

## Experimental section

### Materials

Doxorubicin hydrochloride, dopamine hydrochloride and Cy5 were purchased from Aladdin (Shanghai, China). mPEG-SH, 5000 Da was purchased from Ponsure. Tris-(hydroxymethyl) aminomethane (tris) was obtained from Sangon Biotech (Shanghai, China). Iso-propyl alcohol (IPA, HPLC grade) was purchased from Sinopharm Chemical Reagent. RPMI1640 was purchased from Gibco Life Technologies. Unless otherwise stated, other reagents were of analytical grade.

### Synthesis of Ti@PDA-PEG-DOX NSs

#### Preparation of Ti NSs

Ti nanosheets were prepared using a modified liquid-phase exfoliation of the corresponding bulk sample [17]. First, 200 mg of Ti powder was dispersed in 25 mL of isopropyl alcohol (IPA). Then, the mixture solution was sonicated for 24 h in an ice bath at a power of 520 W, and sonication was set to an on/off cycle of 2 s/2 s. After sonication, the obtained brown dispersions were centrifuged at 6000 rpm for 15 min to discard the unexfoliated Ti powder. Finally, the collected supernatant was centrifuged at 12,000 rpm for 40 min to remove IPA, and the precipitate was dried in a vacuum freeze drier. The dried Ti nanosheets (NSs) were packaged in tin foil to avoid oxidation and stored at 4 °C for further use.

#### Preparation of Ti@PDA-PEG NSs

The PDA layer was obtained on the surface of Ti NSs by oxidation and self-polymerization of dopamine in a weak alkaline solution. Briefly, 5 mg of dopamine hydrochloride was dissolved in 10 mL of Tris–HCl (0.01 M, pH = 8.5) buffer solution, and then 10 mg of Ti NSs was added to the dopamine hydrochloride solution. Afterward, the mixed solution was stirred (250 rpm) in the dark at room temperature overnight. To remove free dopamine, the reaction solution was centrifuged (12,000 rpm, 40 min) and washed using deionized (DI) water.

To graft PEG, 10 mg of Ti@PDA NSs was dispersed in 10 mL DI water with predissolved 30 mg mPEG-SH, and NaOH solution was added to adjust the pH value of the solution to 8.0. After stirring (250 rpm) overnight in the dark at room temperature, the obtained product was centrifuged (12,000 rpm, 40 min) and washed with DI water three times except for unbound mPEG-SH. Subsequently, the Ti@PDA-PEG NS samples were dried in a vacuum freeze drier and stored at 4 °C before use.

#### Drug loading

For DOX loading, the prepared Ti@PDA-PEG NSs (1 mg) were dispersed into phosphate-buffered saline (PBS, pH = 7.4) and then mixed with different doses of free DOX solution to final concentrations of 0.2, 0.4, 0.8 mg/mL at different feeding ratios (DOX/Ti@PDA-PEG NSs feeding ratio = 1.0, 2.0, 4.0). The mixtures were incubated for 24 h in the dark in a shaker at 37 °C and centrifuged at 12,000 rpm for 30 min. DI water washed the precipitate to remove the remaining DOX. Cy5-loaded Ti@PDA-PEG NSs were prepared using a similar method. All the supernatant was collected, and its absorbance at 480 nm was measured by a UV–vis spectrophotometer. The loading efficiency (LE%) of DOX was calculated using the formula below:

$${\text{LE}} \, (\% ) = \frac{{{\text{m}}_{{\text{o}}} - {\text{m}}_{{\text{r}}} }}{{{\text{m}}_{{{\text{Ti}}@{\text{PDA - PEG}}}} }} \times 100\% ,$$

where m_o_ and m_r_ display the mass of the original and residual DOX in solution, respectively.

### Material characterization

The morphology and size of nanosheets were characterized by transmission electron microscopy (TEM, HT7700 Exalens, Hitachi, Japan). Atomic force microscopy (AFM) images were acquired using a Bruker Diension Icon microscope (USA). The size/polydispersity index and zeta potential of the samples were measured on a NanoBrook Omni (NanoBrook Omni, Brookhaven Instruments Co, USA). UV–vis–NIR absorption spectra were collected using a UV–vis–NIR spectrophotometer (UV1800PC, JING HUA Instruments, China). Raman spectra were obtained on a high-resolution confocal Raman microscope (Thermo Fisher DXR 2xi, USA). The chemical compositions of the Ti NSs, Ti@PDA NSs, Ti@PDA-PEG NSs and Ti@PDA-PEG-DOX NSs were analyzed by X-ray photoelectron spectroscopy (XPS, Thermo Scientific ESCALAB 250Xi, USA). Fourier transform infrared spectroscopy (FTIR) was carried out on an IRTracer-100 (Japan) using the KBr method to monitor the synthesis process of the nanosheets.

### Stability evaluation of Ti@PDA-PEG-DOX NSs

For the stability study, Ti@PDA-PEG-DOX NSs (100 μg/mL) were incubated in water, PBS or cell culture medium. The dispersion state of samples in the different media was observed and imaged after 72 h. The particle size and polydispersity index of the Ti@PDA-PEG-DOX NS aqueous solution was measured at predetermined time intervals to study its stability within 72 h. Ti@PDA-PEG-DOX NS aqueous solution was stored at room temperature and protected from light.

### Evaluation of photothermal property

To investigate the effect of the photothermal conditions, the photothermal properties of aqueous solutions of different NSs (Ti NSs, Ti@PDA NSs, Ti@PDA-PEG NSs) at different concentrations (0–200 μg/mL) were measured by exposure to an 808 nm NIR laser (Beijing Laserwave Electronic Technology Co., Ltd., Beijing, China) at a power density of 1.1 W/cm^2^ for 10 min. To study the influence of power density on photothermal performance, Ti@PDA-PEG NS solution (100 μg/mL) was irradiated under an NIR laser at different power densities (0.5–1.5 W/cm^2^). In addition, the Ti@PDA-PEG NS solution (100 μg/mL) was irradiated using an 808 nm laser on and off for five cycles to assess their photothermal stability. The temperature changes of the different aqueous solutions were recorded using an infrared thermal camera (HT-H8 Infrared Camera, XINTEST).

### In vitro pH-responsive and NIR-responsive release of DOX from Ti@PDA-PEG-DOX NSs

To assess the DOX release profile of Ti@PDA-PEG-DOX NSs, 4 mL of Ti@PDA-PEG-DOX NSs solution was transferred a dialysis bag (MWCO = 8–14 kDa), and merged in 36 mL PBS at one of two different pH values (pH = 7.4 or 5.5).The in vitro release experiment was carried out in the shaker (37 °C, 120 rpm) for 72 h. During the incubation process, 4 mL of outside release media was removed at different time points to detect absorbance at 480 nm to determine the amount of released drug, and fresh PBS solution of the same volume and pH value was added. To investigate NIR-triggered release, the experiments were carried out at pH = 5.5 and irradiated with an 808 nm NIR laser at a power density of 1.1 W/cm^2^ over a period of 5 min.

### Cellular experiments

#### Cell culture

The 4T1 cell line was cultured in RPMI 1640 culture medium supplemented with antibiotics penicillin (100 U/mL) and streptomycin (100 μg/mL) containing 10% (v/v) fetal bovine serum. Cells were incubated in a 5% CO_2_-humidified atmosphere at 37 °C.

#### In vitro biocompatibility assay of Ti@PDA-PEG NSs

The cytobiocompatibility of Ti@PDA-PEG NSs to cells was determined by Cell Counting Kit-8 (CCK-8) assay. First, the prepared 4T1 cell suspension was inoculated at a density of 5 × 10^4^ into a 96-well plate and cultured in an atmosphere containing 5% CO_2_ at 37 °C for 24 h. Subsequently, the earlier medium was replaced with 100 µL of fresh medium containing different concentrations of Ti@PDA-PEG NSs (0–100 µg/mL) and incubated for an additional 24 h. Then, 10 µL CCK-8 solution was added to each well, and the whole plate was incubated for another 2 h. Finally, the absorption of each well was measured at 450 nm using an enzyme labeling instrument, and the cell viability was calculated by comparison with a control group using the following equation.

$${\text{Cell }}\;{\text{viability}} (\% ) = \frac{{{\text{OD}}_{{{\text{sample}}}} - {\text{OD}}_{{{\text{blank}}}} }}{{{\text{OD}}_{{{\text{control}}}} - {\text{OD}}_{{{\text{blank}}}} }} \times 100\% .$$

#### In vitro evaluation of cell cytotoxicity

To evaluate the toxic effect of Ti@PDA-PEG-DOX NSs in vitro, 4T1 cells were cultured in RPMI 1640 medium. The treatment groups included (1) the control group, (2) NIR irradiation group, (3) Ti@PDA-PEG NSs group, (4) Ti@PDA-PEG NSs + NIR irradiation group, (5) free DOX group, (6) Ti@PDA-PEG-DOX NSs group and (7) Ti@PDA-PEG-DOX NSs + NIR irradiation group. The DOX concentration was equivalent to 50 μg/mL, and the Ti@PDA-PEG NS concentration was approximately 40 µg/mL. 4T1 cells (cell density of 5 × 10^4^) were seeded in 96-well plates at a dose of 100 μL per well and cultured for 24 h. Then, the culture medium was discarded, and new medium containing samples was added to the 96-well plate according to the different groups. Group 2, Group 4 and Group 7 were irradiated with an 808 nm NIR laser (1.1 W/cm^2^) for 5 min. After incubation for 18 h, the percentage of cell viability was determined by CCK-8 analysis as described above.

#### Cellular uptake

To study the cellular uptake of Ti@PDA-PEG-DOX NSs using a fluorescence microscope. A total of 5 × 10^4^ 4T1 cells were seeded into 6-well plates and incubated for 24 h. Then, the culture medium was replaced with fresh media containing Ti@PDA-PEG-DOX NSs (Ti@PDA-PEG NS concentration: ~ 40 µg/mL, DOX concentration: ~ 50 µg/mL). After 5 min incubation with/without NIR laser irradiation (808 nm, 1.1 W/cm^2^), the 6-well plate was placed in a 37 °C, 5% CO_2_ incubator for 4 h. Afterward, the cells were washed three times with PBS, fixed using 4% paraformaldehyde solution and stained with DAPI for nuclear staining. Then, the cells were rinsed again with PBS and imaged using a fluorescence microscope.

### In vivo antitumor study

#### Establishment of tumor models

Healthy female mice aged 5–6 weeks (approximately 18 g, BALB/c) were purchased and fed in a standard laboratory. All animal experimental procedures were approved by the Animal Ethics Committee of China Pharmaceutical University. To establish the tumor models, 4T1 cell suspensions (100 μL) in PBS at a density of 7 × 10^6^ cells were injected subcutaneously into the armpit of the right limb. The tumor sizes were measured daily using a digital Vernier caliper to quantify tumor growth. When the average tumor volume of the mice reached approximately 150 mm^3^, drug delivery to the mice was carried out. The tumor volume of the mice was calculated by the formula: V = 0.5 × L × W^2^ (V: volume, L: tumor length, W: tumor width).

#### In vivo fluorescence imaging and biodistribution analysis

To investigate the in vivo biodistribution, 4T1 tumor-bearing female mice were given an intravenous injection of Cy5-loaded Ti@PDA-PEG NSs (the dosage of Cy5 was equivalent to 1 mg/kg for each mouse) via the tail vein. At fixed time points (1, 2, 4, 8, 12, and 24 h postinjection), in vivo fluorescence imaging experiments were performed using the IVIS Lumina III In Vivo Imaging System (PerkinElmer, USA) after anesthetizing the mice with 10% chloral hydrate solution. Finally, the mice were sacrificed, and major organs, including the heart, liver, spleen, lung, kidney and tumor tissue, were excised and imaged immediately.

#### Infrared thermal imaging

To investigate the effect of photothermal application in vivo, BALB/c mice bearing 4T1 tumors were utilized as the animal model for the experiments. 4T1 tumor-bearing mice were injected intravenously with normal saline (NS) and Ti@PDA-PEG-DOX NSs (the dosage of Ti@PDA-PEG NSs ~ 5 mg/kg, the dosage of DOX ~ 6 mg/kg). Twenty-four hours post administration [24, 25], the mice were anesthetized, and the tumor sites were irradiated with an 808 nm laser for 5 min (power density of 1.1 W/cm^2^). During irradiation, photothermal images of the tumor site were recorded by an IR camera (HT-H8 Infrared Camera, XINTEST) at a fixed time interval.

#### In vivo antitumor study

When the tumor volume reached approximately 150 mm^3^, 4T1 tumor-bearing mice were randomly assigned into five groups (5 mice per group): (1) normal saline (NS) group, (2) Ti@PDA-PEG NSs + NIR irradiation group, (3) DOX group, (4) Ti@PDA-PEG-DOX NSs group, and (5) Ti@PDA-PEG-DOX NSs + NIR irradiation group. The dose was ~ 6 mg/kg in terms of DOX, and the dose was ~ 5 mg/kg in terms of Ti@PDA-PEG NSs. The tumor sites of Group 2 and Group 5 were irradiated by an 808 nm laser (1.1 W/cm^2^, 5 min) at 24 h postinjection. Groups 1, 3 and 4 were injected intravenously with 100 μL of sample solution on treatment Days 0, 4 and 8, and only one treatment was performed on Day 0 for Groups 2 and 5. The tumor volume and body weight of the treated mice were recorded at 2-day intervals for 14 days. At the end point of the animal experiment, the mice were euthanized. The tumors and the main organs (heart, liver, spleen, lung, kidney) were collected for further studies (H&E and TUNEL assay).

### Statistical data analysis

The experimental data are shown as the mean ± standard deviation (n = 3), and the experimental results were analyzed by STATISTICA 6.0. Statistical significance was assigned to *p < 0.05, **p < 0.01 and ***p < 0.001 as extreme statistical significance.

## Results and discussion

### Synthesis and characterizations of Ti@PDA-PEG-DOX NSs

**Scheme 1:**
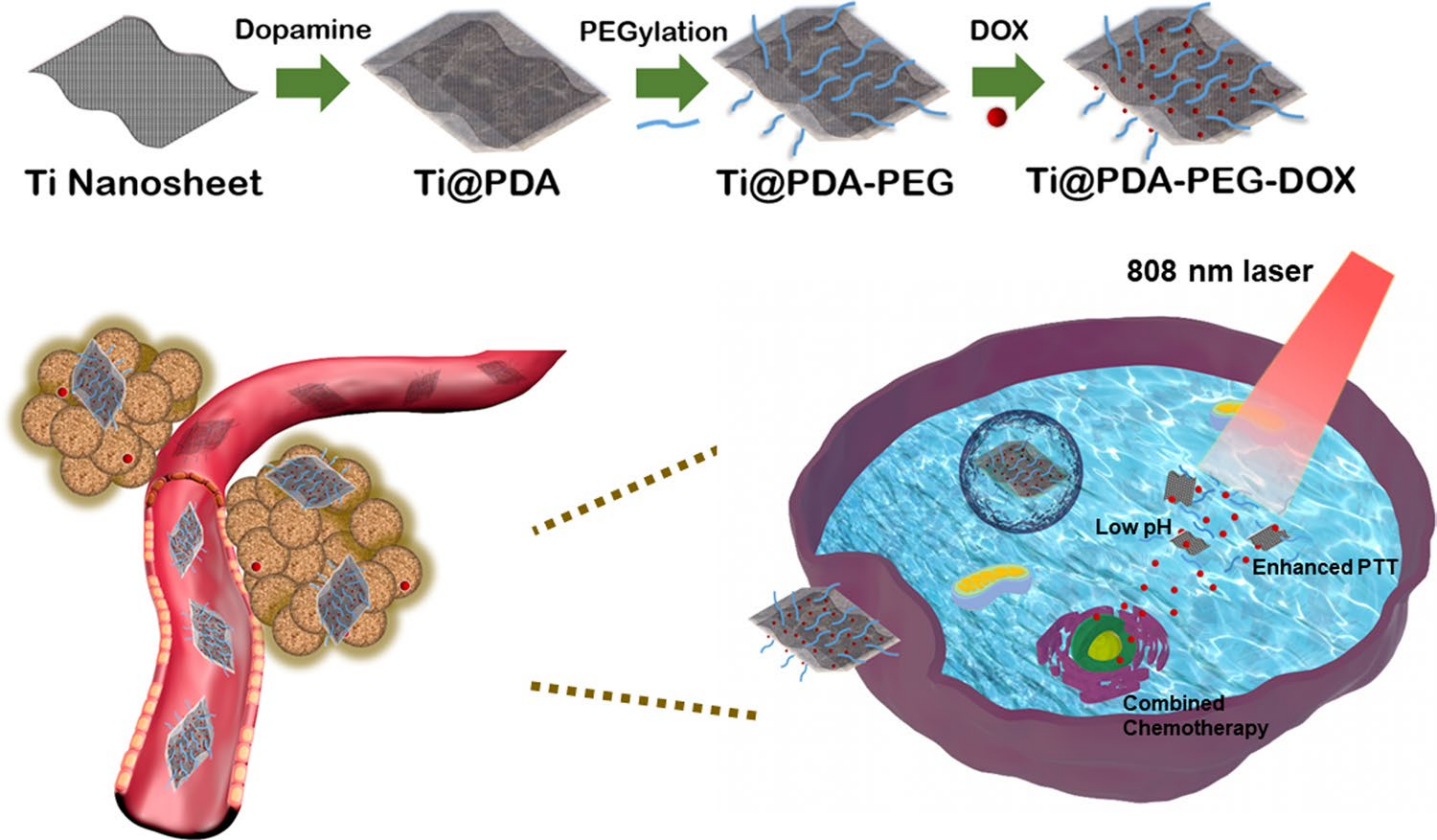
Schematic illustration of the synthesis of a multifunctional Ti@PDA-PEG-DOX nanosheet drug delivery system and the combined photothermo-chemotherapy of tumor cells

The synthetic process of Ti@PDA-PEG-DOX NSs is shown in Scheme 1. First, according to the previously reported method [17], Ti NSs were prepared using a modified liquid exfoliation technique from bulk Ti in IPA. Afterward, a thin PDA film was modified on the surface of Ti NS through oxidative self-polymerization of dopamine in Tris buffer (pH = 8.5). The PDA shell improved the stability of bare Ti and provided an ideal platform for the secondary modification of NSs. Then, mPEG-SH was introduced to the layer of PDA by a Michael addition reaction to further improve the behaviors of Ti@PDA NSs. Finally, DOX was loaded into Ti@PDA-PEG NSs via π–π stacking [26], hydrophobic interactions [27], electrostatic interactions [28, 29], or the interaction between DOX (–NH_2_) and the functional moieties of PDA (quinone-like structure) [30], which achieved ultrahigh DOX loading. At our test dose, the highest DOX loading capacity of Ti@PDA-PEG NSs reached 276% (Additional file 1: Fig. S1).

**Fig. 1:**
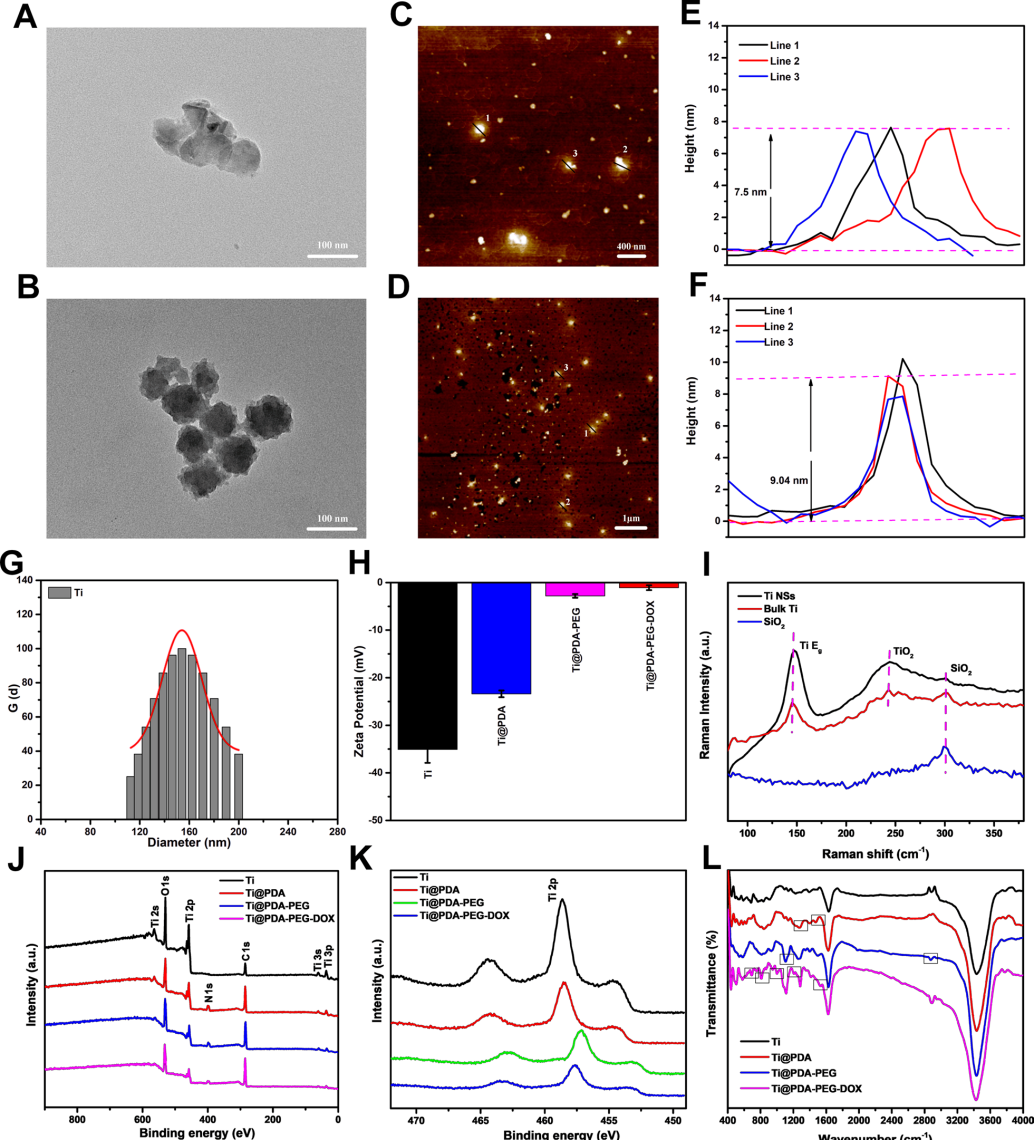
**A**, **B** TEM image of Ti NSs and Ti@PDA NSs. **C**, **D** AFM image of Ti NSs and Ti@PDA NSs. **E**, **F** Thickness measured from **C** and **D**. **G** Size distribution of the Ti NSs by DLS. **H** Zeta potentials of Ti NSs, Ti@PDA NSs, Ti@PDA-PEG NSs and Ti@PDA-PEG-DOX NSs. **I** Raman spectra acquired from the Ti NSs and bulk Ti. **J**, **K** XPS spectra of Ti NSs, Ti@PDA NSs, Ti@PDA-PEG NSs and Ti@PDA-PEG-DOX NSs, **J** survey spectrum. **K** Ti 2p spectrum. **L** FT-IR spectra of the Ti-based NSs

The morphology and size of Ti NSs, Ti@PDA NSs, Ti@PDA-PEG NSs and Ti@PDA-PEG-DOX NSs were observed using a transmission electron microscope (TEM, Fig. 1A, B and Additional file 1: Fig. S2). The liquid-exfoliated Ti NSs showed an ultrathin nanosheet morphology and an average size of ≈ 100 nm in TEM images. The thickness of Ti NSs and Ti@PDA NSs was determined by atomic force microscopy (AFM, Fig. 1C–F). The thickness of Ti NSs was approximately 7 nm, and the thickness of Ti@PDA increased to approximately 9 nm because of the PDA coating on the Ti NS surface. Dynamic light scattering (DLS) analysis results (Fig. 1G and Additional file 1: Fig. S3) indicated that Ti NSs were dispersed in water with a hydrodynamic diameter of approximately 150 nm, which was larger than the size of the nanosheets obtained from TEM. These results were mainly due to different sample states involved in the measurement processes. The hydration radius of the nanoparticles or the hydrodynamic diameter (hydrated state) of the aggregate after the agglomeration of nanoparticles were measured by DLS, whereas TEM often gives the size of the dried nanosheets [31, 32].

The zeta potential of different samples was investigated (Fig. 1H). Bare Ti NSs had a zeta potential of − 35 mV, and the zeta potential of Ti@PDA NSs increased to − 23 mV. This change in value could be attributed to the deprotonation of phenolic hydroxyl groups of the PDA shell at neutral pH [33]. When Ti@PDA-PEG NSs were loaded with DOX, their zeta potential was − 1.1 mV due to the positively charged amino groups on DOX [34]. A slightly negative charge would be fit for cell accessibility [35]. The absorption spectra showed that Ti NSs and Ti@PDA-PEG-DOX NSs had broad and strong absorption bands covering the UV to NIR regions (Additional file 1: Fig. S4). To use the NIR transparent window between 700 and 1000 nm of biological tissues for PTT, strong absorptance in the NIR region is necessary [36]. The Ti@PDA-PEG-DOX NS solution exhibited a strong UV–vis absorption peak at approximately 480 nm, further confirming the successful modification of DOX molecules.

Raman spectroscopy was performed to characterize the bulk Ti and exfoliated Ti NSs (Fig. 1I), which showed similar Raman peaks. For bulk Ti, the E_g_ peak at ≈ 145.9 cm^−1^ was observed, which is a well-known Eg mode of *hcp* titanium [37]. A peak at ≈ 147.5 cm^−1^ of the exfoliated Ti NSs implies a slight blueshift for the ultrathin Ti NSs. The peaks at ~ 242 cm^−1^ for both bulk Ti and exfoliated Ti NSs were caused by the rocking vibration of the titanium-oxygen (Ti–O) bond of rutile TiO_2_ [38]. The background signal of the silicon substrate (with the peak at approximately 300 cm^−1^) could be observed for all samples. The result shows that the structure of exfoliated Ti NSs was the same as bulk Ti.

The chemical components of the nanosheets were analyzed by X-ray photoelectron spectroscopy (XPS, Fig. 1J, K). Compared with the spectra of the bare Ti NSs, the N1s peak at a binding energy of ∼ 400 eV in the spectra of Ti@PDA NSs and Ti@PDA-PEG NSs was observed, which confirmed the presence of a PDA layer. Furthermore, the Ti 2p peak intensity of Ti, Ti@PDA, Ti@PDA-PEG and Ti@PDA-PEG-DOX NSs showed a gradual decreasing trend, while the intensity of C1s gradually increased. These results implied that the corresponding compounds had been successfully modified. The low C ls peak in the spectra of Ti NSs may be attributed to environmental pollution. The O 1 s peak at ~ 530 eV in the bare Ti NSs corresponds to the O^2−^ binding energy of Ti–OH groups [39], which may be due to Ti–O combined in solution or Ti NS oxidation in air. In Fig. 1K, a shift of Ti 2p peaks in the spectra indicated that the chemical states of the samples changed from an oxide state to a suboxide state with the modification of nanosheets [40, 41], which further demonstrated that the modification could improve the stability of pure Ti NSs.

Figure 1L shows the Fourier transform infrared spectra (FT-IR) of Ti, Ti@PDA, Ti@PDA-PEG and Ti@PDA-PEG-DOX NSs. The adsorption peak at ~ 3437 cm^−1^ could be ascribed to the O–H stretching mode. The –OH group was produced by Ti–O [42, 43], and the presence of Ti–O on the surface of Ti NSs was confirmed by Raman measurements and XPS. After surface coating with the PDA layer, a peak at ~ 1477 cm^−1^ was displayed, which was due to the skeleton vibration from the benzene ring of PDA. The peaks at ~ 1274 cm^−1^ can be assigned to C–N stretching vibrations. For the PEGylated Ti@PDA NSs, the absorption peak at ∼ 2876 cm^−1^ was assigned to the C–H stretching vibration mode from CH_2_ of PEG, and the adsorption band at ~ 1108 cm^−1^ could be ascribed to the C–O stretching mode. Fourier transform infrared (FTIR) spectroscopy of Ti@PDA-PEG-DOX NSs showed several absorption signals of DOX. The characteristic adsorption peaks at approximately 692 cm^−1^, 809 cm^−1^, 995 cm^−1^, 1285 cm^−1^ and 1528 cm^−1^ can be assigned to the weak deformation vibration of the benzene ring [44], the vibration of the single oxygen six-membered ring, the C–C–C bending vibration of the ketone group, the C–H vibration of the single oxygen six-membered ring and the C–C vibration of the benzene ring, respectively [45]. The FT-IR results indicated that the Ti@PDA-PEG-DOX NS nanoplatform was synthesized successfully.

### Stability study of Ti@PDA-PEG-DOX NSs

Ti@PDA-PEG-DOX NSs were excellently stable in vitro within 72 h when incubated in water, phosphate buffered saline (PBS, pH = 7.4), or RPMI 1640 medium containing 10% fetal bovine serum (FBS) (Additional file 1: Fig. S5), and the color and dispersion of the test solution remained unchanged. This was further confirmed by the hydrodynamic diameter of the Ti@PDA-PEG-DOX NS aqueous solution without changing for 72 h (Additional file 1: Fig. S6).

### Evaluation of photothermal performance

**Fig. 2:**
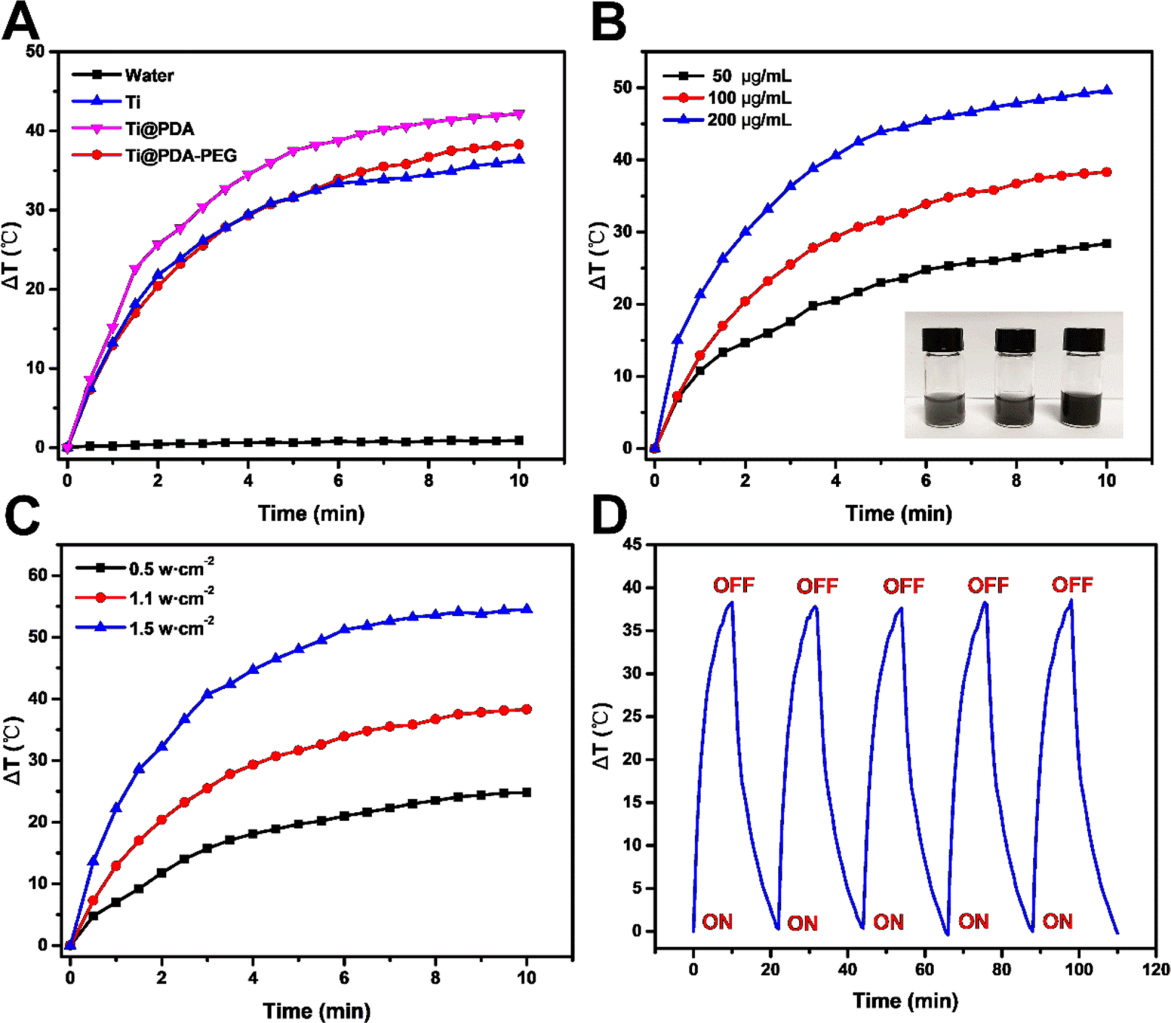
**A** Temperature elevation curves of water, Ti, Ti@PDA, and Ti@PDA-PEG solutions under 808 nm laser irradiation (1.1 W/cm^2^). **B** Temperature increase of different concentrations of Ti@PDA-PEG NSs upon irradiation (808 nm, 1.1 W/cm^2^). **C** Photothermal heating curves of 100 μg/mL Ti@PDA-PEG NS solution under various power intensities. **D** Temperature alternation of Ti@PDA-PEG NSs (100 μg/mL) solution under irradiation at 808 nm at a power density of 1.1 W/cm^2^ for five laser on/off cycles

The photothermal efficiency of Ti-based NSs was further investigated in vitro by recording the temperature changes under irradiation with an 808 nm NIR laser. As previously reported [46], 808 nm laser has the highest equilibrium point among tissue penetration, tissue self-heating, and photothermal conversion of photothermal agents, which is the main reason that 808 nm laser is preferred for evaluating photothermal conversion efficiency. As depicted in Fig. 2A, for water and aqueous solutions of Ti NSs, Ti@PDA NSs, Ti@PDA-PEG NSs, temperature increased by 0.9 °C, 36.3 °C, 38.3 °C and 42.2 °C, respectively upon NIR laser irradiation (808 nm, 1.1 W/cm^2^, 10 min). The results showed that Ti-based NSs had amazing photothermal conversion capability. Moreover, the Ti@PDA NS solution had the highest temperature increase among the four samples, suggesting that the PDA coating also has photothermal conversion efficiency. In addition, the temperature of Ti@PDA-PEG NSs followed a dose-dependent and laser power-dependent relationship (Fig. 2B, C). According to pioneering reports, 43.0 °C is the critical temperature to induce the death of tumor cells [47, 48]. From the results of photothermal performance research, Ti@PDA-PEG NSs could easily achieve a critical temperature or higher temperature at low concentrations or short-term laser irradiation. As shown in Fig. 2D, the stability of the photothermal effect was assessed using an 808 nm laser (power density of 1.1 W/cm^2^) on/off for 5 cycles. The highest temperature of Ti@PDA-PEG NSs had no significant change, revealing the excellent photothermal stability of the nanocarrier. Taken together, this evidence indicated that Ti@PDA-PEG NSs are highly promising PTAs.

### In vitro pH-responsive and NIR-responsive drug release

**Fig. 3:**
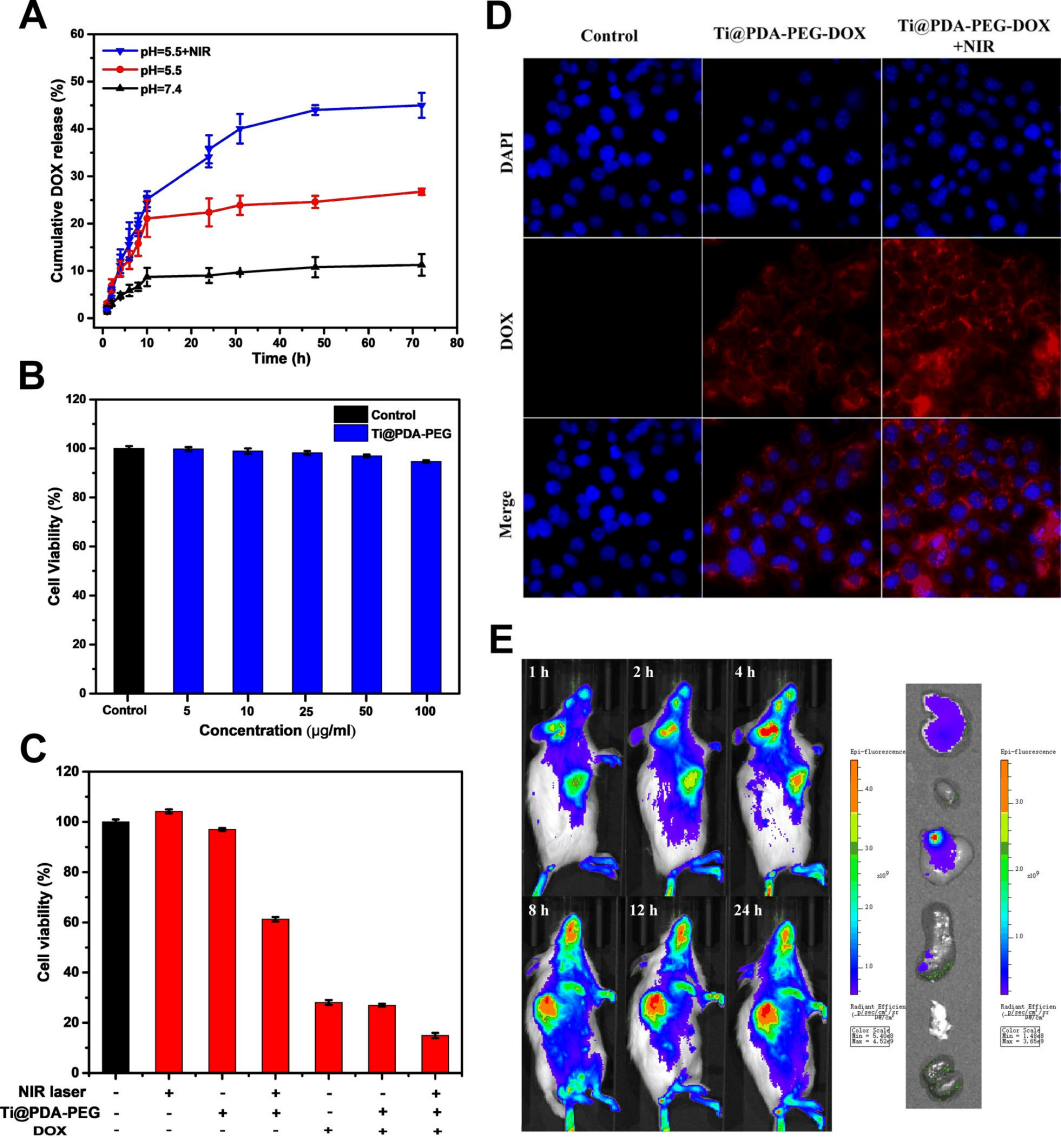
**A** DOX release from Ti@PDA-PEG-DOX NSs in vitro at different pH values with/without an 808 nm NIR laser (1.1 W/cm^2^, 5 min). **B** Effect of different concentrations of Ti@PDA-PEG NSs on cell viability. **C** Cell viability of 4T1 cells with different treatments. **D** Fluorescence images of 4T1 cells treated with Ti@PDA-PEG-DOX NSs with/without laser irradiation for 5 min (original magnification, ×400). **E** In vivo fluorescence imaging of 4T1 tumor-bearing mice at different time points that received Cy5-labeled Ti@PDA-PEG NSs via tail intravenous injection and ex vivo fluorescence images of major organs and tumors after systemic administration at 24 h

The drug-release performance of Ti@PDA-PEG-DOX NSs was evaluated under different pH values (pH 7.4 and pH 5.5) with or without 808 nm laser irradiation (Fig. 3A). Within 72 h, only approximately 11.3% of DOX was released from the Ti@PDA-PEG-DOX NSs at pH 7.4, while the release of DOX was elevated to approximately 26.7% at pH 5.5 over the same time period. The pH-dependent DOX release pattern may be attributed to the gradual degradation of the PDA layer and the increased protonation of the amino group in DOX molecules in an acidic solution [30]. Given the acidic microenvironments of tumors and intracellular acidic endosomes and lysosomes [49], this pH-responsive release of drugs will benefit the application of Ti@PDA-PEG-DOX NSs for tumor therapy. After NIR laser irradiation for 5 min at certain time intervals at pH 5.5, the accumulated release of DOX was increased to 45.0% within 72 h, indicating that NIR triggered accelerated DOX release by Ti@PDA-PEG-DOX NSs [50]. NIR-responsive accelerated DOX release could be ascribed to the photothermal property of Ti@PDA-PEG NSs, which made the local temperature rise under irradiation weaken the interaction between DOX and the drug carrier. In short, the pH-responsive and NIR-responsive drug release behavior revealed that the Ti@PDA-PEG-DOX NS drug-delivery nanoplatform was an excellent candidate for antitumor therapy.

### In vitro cytotoxicity assessment

The biocompatibility of Ti@PDA-PEG NSs for 4T1 cells was evaluated by CCK-8 assay in vitro. As Fig. 3B shows, when the concentration of Ti@PDA-PEG NSs was as high as 100 μg/mL, the viability of 4T1 cells remained above 94%. The results suggest that Ti@PDA-PEG NSs have excellent biocompatibility as drug carriers.

4T1 cells were incubated with different samples with or without NIR irradiation (808 nm, 1.1 W/cm^2^, 5 min) for 24 h to study the cytotoxicity of NSs in vitro. As shown in Fig. 3C, the cell viability of the NIR group and Ti@PDA-PEG NSs group was similar to that of the negative control group, indicating negligible cytotoxicity using laser irradiation alone or Ti@PDA-PEG NSs alone in vitro. In addition, Ti@PDA-PEG-DOX NSs showed a slightly higher inhibition rate than free DOX at the same DOX concentration level, which may be attributed to the responsive release of DOX [30]. Notably, when the Ti@PDA-PEG NS group was exposed to laser irradiation, the viability of 4T1 cells was decreased to only 61%. While the Ti@PDA-PEG NS group was combined with both DOX loading and NIR laser irradiation, the growth of cells was obviously inhibited (15%). The results demonstrate that PTT combined with chemotherapy is better than the sole therapy effect and that Ti@PDA-PEG-DOX NSs have great potential as multifunctional nanoplatforms for enhancing tumor therapeutic effects.

### Cellular uptake

The cellular uptake of the control group, Ti@PDA-PEG-DOX NSs group, and Ti@PDA-PEG-DOX NSs + NIR irradiation group was observed in vitro by fluorescence microscopy (Fig. 3D). The blue and red fluorescence signals are from the DAPI-stained nuclei and DOX, respectively. The red fluorescence in both the cytoplasm and nucleus of the Ti@PDA-PEG-DOX NS group with irradiation was stronger than that of the Ti@PDA-PEG-DOX NS group without irradiation at the same incubation time. This is because the local temperature rises under radiation accelerated the release of DOX from Ti@PDA-PEG-DOX NSs [51, 52]. No red fluorescence signal in the picture of the control group was observed.

### In vivo imaging and biodistribution analysis

After tail vein injection, the biodistribution and tumor accumulation of Cy5-labeled Ti@PDA-PEG NSs were monitored in 4T1 tumor-bearing mice at determined time points using an in vivo imaging system (Fig. 3E). As time passed, the fluorescence signals of the tumor sites gradually increased. Meanwhile, the fluorescence signals at the tumor sites remained very strong even 24 h after injection, which was attributed to the prolonged retention of NSs in the tumor via blood circulation and the enhanced permeability and retention (EPR) effect [53]. The right side of Fig. 3E shows an ex vivo fluorescence image of excised main organs (heart, liver, spleen, lung, kidney) and tumor tissue after the experiment was finished. Fluorescence signals were observed in the liver, tumor and spleen, and the higher fluorescence signals in the liver may be due to the absorption of NSs by the reticuloendothelial system. The overall results demonstrated the promise of Ti@PDA-PEG-DOX NSs for systematic delivery in tumor treatment.

### In vivo photothermal effects

**Fig. 4:**
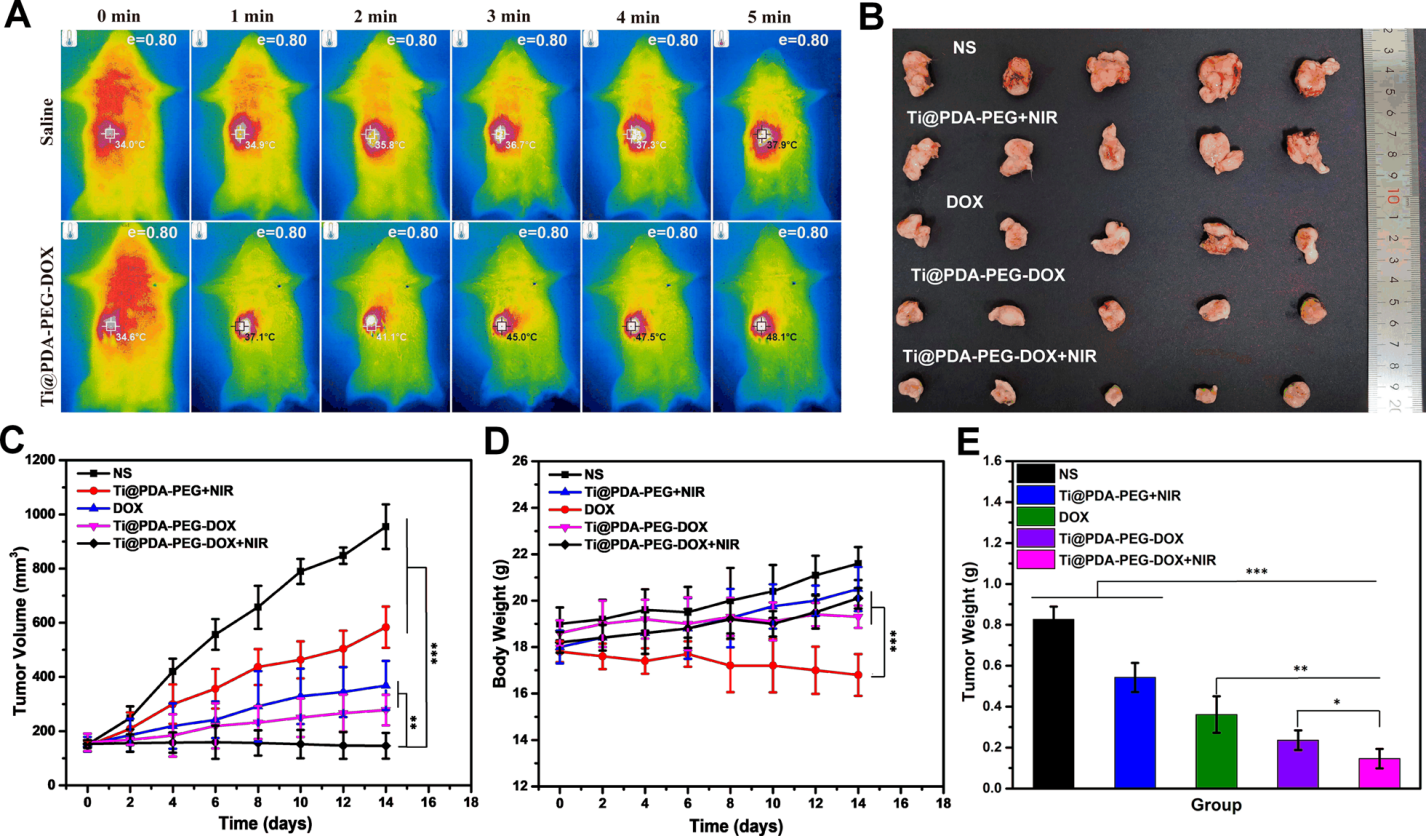
**A** In vivo infrared thermal images. **B** Images of tumors in each group removed from the sacrificed mice at the end point of the study. **C**, **D** Tumor volume growth curve and body weight change curve of 4T1 tumor-bearing mice after intravenous injection with normal saline (NS), Ti@PDA-PEG NSs, free DOX, Ti@PDA-PEG-DOX NSs and Ti@PDA-PEG-DOX NSs with or without NIR laser irradiation. **E** Tumor weight of each group taken from the sacrificed mice after the experiment was finished. (*P < 0.05, **P < 0.01, ***P < 0.001)

As shown in Fig. 4A, the tumor surface temperatures of the mice increased from approximately 34.6 °C to 48.1 °C within 5 min under NIR irradiation (808 nm, 1.1 W/cm^2^) after intravenous injection of Ti@PDA-PEG-DOX NSs, owing to the excellent photothermal effects of Ti@PDA-PEG NSs. Elevated temperature at the tumor surface was enough to trigger drug release based on the in vitro studies and even led to tumor ablation [54, 55]. In contrast, the mice treated with normal saline (NS) did not show obvious temperature changes at the tumor site after laser irradiation. In summary, Ti@PDA-PEG-DOX NSs are outstanding photothermal conversion capacity in vivo.

### In vivo antitumor study

The antitumor efficacy was assessed in a 4T1 tumor-bearing BALB/c mouse model. As depicted in Fig. 4B, C and E, the tumors of the normal saline (NS) group grew fastest and formed the largest solid tumor. The tumor tissue of the Ti@PDA-PEG NSs + NIR irradiation group also grew rapidly due to the poor antitumor effect of a single photothermal treatment. As we expected, the tumor volume of the Ti@PDA-PEG-DOX NSs group was better than that of the free DOX group. Particularly, as evidenced by the smallest tumor volume, the group treated with Ti@PDA-PEG-DOX NSs + NIR laser irradiation had the best antitumor effect. This was ascribed to the fact that the EPR effect led to more accumulation of NSs, while the heat produced by NIR irradiation could induce the release of DOX from Ti@PDA-PEG-DOX NSs and promote the endocytosis of nanomaterials [56]. Consequently, the results soundly implied a satisfactory treatment effect of Ti@PDA-PEG-DOX NSs combined with NIR irradiation on tumor development.

The mice were weighed during the whole experiment to evaluate the safety of each treatment plan. As shown in Fig. 4D, the trend of change in the body weight in the free DOX group showed a downtrend compared with the other groups, reflecting that the free DOX formulation had a toxic effect. The mice in the other groups showed slight increases or slight fluctuations in body weight throughout the treatment period, indicating the safety and very low toxicity of loading DOX onto Ti@PDA-PEG NSs. This finding supported that the Ti@PDA-PEG-DOX NS drug delivery system is more biosafe than free DOX.

**Fig. 5:**
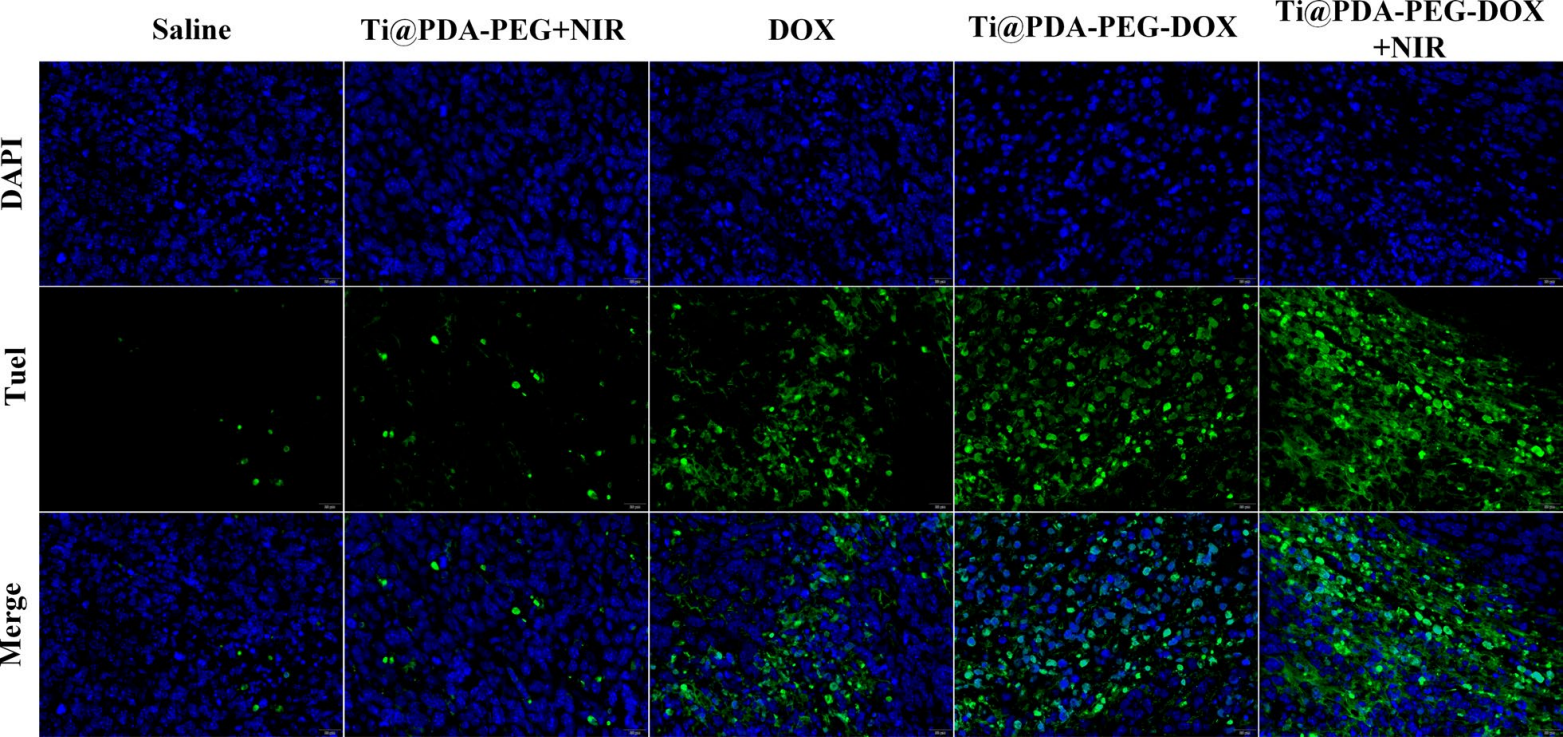
TUNEL staining of tumor tissues after various treatments for 14 days, blue—nuclei and green—TUNEL (scale bar = 20 μm)

The antitumor effect of NSs was further investigated by immunohistochemical analysis (H&E and TUNEL assays). The apoptosis of tumor cells in the tumor tissue was assessed by a TUNEL detection kit (green fluorescence). The nuclei and broken DNA of tumor cells were labeled with blue fluorescence and green fluorescence, respectively, after staining. As shown in Fig. 5, compared with other groups, the degree of apoptosis in the Ti@PDA-PEG-DOX NSs + NIR irradiation group was the most serious. H&E staining was used to observe the physiological morphology of the major organs of mice (Additional file 1: Fig. S7). Hepatocellular edema could be observed in the free DOX group due to side effects of DOX, which also explains the reason for the weight loss of mice in the free DOX group. In addition, the histomorphology of major organs revealed no obvious pathologic changes in the other groups. The experimental results further confirm that Ti@PDA-PEG-DOX NS + NIR irradiation can effectively inhibit tumor growth and has high biosafety in vivo.

## Conclusions

In summary, we successfully fabricated Ti@PDA-PEG-DOX NSs as a drug-delivery system for combined photochemo tumor therapy. These Ti@PDA-PEG NSs showed high drug loading capacity and excellent photothermal conversion efficiency for PTT. Using DOX as a model chemotherapy drug, the Ti@PDA-PEG-DOX NSs displayed pH-responsive and photo-responsive release properties after either extrinsic or intrinsic stimuli. According to the results, the Ti@PDA-PEG-DOX NSs nanoplatform exhibited biosafety, biocompatibility and antitumor effects both in vitro and in vivo. The multifunctional platform based on Ti NSs has shown favorable prospects in the application of tumor combination therapy.

## Supplementary Information

**Additional file 1: Figure S1.** DOX loading efficiencies on Ti@PDA-PEG NSs (%) with increasing DOX feeding concentrations. **Figure S2.** (A, B) TEM images of Ti@PDA-PEG NSs, and (C, D) TEM images of Ti@PDA-PEG-DOX NSs. **Figure S3.** DLS size distribution of different samples. **Figure S4.** UV–vis–NIR absorption spectra of Ti and Ti@PDA-PEG-DOX NSs. **Figure S5.** Photo images of the Ti@PDA-PEG-DOX NSs incubated in water, PBS and cell culture. **Figure S6.** Stability of Ti@PDA-PEG-DOX NSs in water during 72 h. **Figure S7.** Histology analysis of the major organs extracted from tumor bearing mice stained with hematoxylin and eosin (H&E) after treatment with saline, Ti@PDA-PEG NSs + NIR, free DOX,Ti@PDA-PEG-DOX NSs, Ti@PDA-PEG-DOX NSs + NIR for 14 days (scale bar = 50 μm).

## References

[1] Yang Z, Gao D, Guo X, Jin L, Zheng J, Wang Y, Chen S, Zheng X, Zeng L, Guo M (2020). Fighting immune cold and reprogramming immunosuppressive tumor microenvironment with red blood cell membrane-camouflaged nanobullets. ACS Nano.

[2] Zhang L, Zhang Y, Xue Y, Wu Y, Wang Q, Xue L, Su Z, Zhang C (2019). Transforming weakness into strength: photothermal-therapy-induced inflammation enhanced cytopharmaceutical chemotherapy as a combination anticancer treatment. Adv Mater.

[3] Zha Z, Zhang S, Deng Z, Li Y, Li C, Dai Z (2013). Enzyme-responsive copper sulphide nanoparticles for combined photoacoustic imaging, tumor-selective chemotherapy and photothermal therapy. Chem Commun.

[4] Gao D, Shi Y, Ni J, Chen S, Wang Y, Zhao B, Song M, Guo X, Ren X, Zhang X (2021). NIR/MRI-guided oxygen-independent carrier-free anti-tumor nano-theranostics. Small.

[5] Wang Q, Dai Y, Xu J, Cai J, Niu X, Zhang L, Chen R, Shen Q, Huang W, Fan Q (2019). All-in-one phototheranostics: single laser triggers NIR-II fluorescence/photoacoustic imaging guided photothermal/photodynamic/chemo combination therapy. Adv Func Mater.

[6] Li B, Wang Y, He J (2019). Gold nanorods-based smart nanoplatforms for synergic thermotherapy and chemotherapy of tumor metastasis. ACS Appl Mater Interfaces.

[7] Novoselov KS, Geim AK, Morozov SV, Jiang D, Zhang Y, Dubonos SV, Grigorieva IV, Firsov AA (2004). Electric field effect in atomically thin carbon films. Science.

[8] Guo Y, Huang J, Fang Y, Huang H, Wu J (2022). 1D, 2D, and 3D Scaffolds promoting angiogenesis for enhanced wound healing. Chem Eng J.

[9] Naguib M, Kurtoglu M, Presser V, Lu J, Niu J, Heon M, Hultman L, Gogotsi Y, Barsoum MW (2011). Two-dimensional nanocrystals produced by exfoliation of Ti_3_ AlC_2_. Adv Mater.

[10] Churchill HO, Jarillo-Herrero P (2014). Two-dimensional crystals: phosphorus joins the family. Nat Nanotechnol.

[11] Chhowalla M, Liu Z, Zhang H (2015). Two-dimensional transition metal dichalcogenide (TMD) nanosheets. Chem Soc Rev.

[12] Zhuang Y, Han S, Fang Y, Huang H, Wu J (2022). Multidimensional transitional metal-actuated nanoplatforms for cancer chemodynamic modulation. Coord Chem Rev.

[13] Liu S, Pan X, Liu H (2020). Two-dimensional nanomaterials for photothermal therapy. Angew Chem Int Ed Engl.

[14] Li L, Kim J, Jin C, Ye GJ, Qiu DY, da Jornada FH, Shi Z, Chen L, Zhang Z, Yang F (2017). Direct observation of the layer-dependent electronic structure in phosphorene. Nat Nanotechnol.

[15] Ji X, Kong N, Wang J, Li W, Xiao Y, Gan ST, Zhang Y, Li Y, Song X, Xiong Q (2018). A novel top-down synthesis of ultrathin 2D boron nanosheets for multimodal imaging-guided cancer therapy. Adv Mater.

[16] Liu G, Zou J, Tang Q, Yang X, Zhang Y, Zhang Q, Huang W, Chen P, Shao J, Dong X (2017). Surface modified Ti_3_C_2_ MXene nanosheets for tumor targeting photothermal/photodynamic/chemo synergistic therapy. ACS Appl Mater Interfaces.

[17] Xie Z, Chen S, Duo Y, Zhu Y, Fan T, Zou Q, Qu M, Lin Z, Zhao J, Li Y (2019). Biocompatible two-dimensional titanium nanosheets for multimodal imaging-guided cancer theranostics. ACS Appl Mater Interfaces.

[18] Wang B, Wang JH, Liu Q, Huang H, Chen M, Li K, Li C, Yu XF, Chu PK (2014). Rose-bengal-conjugated gold nanorods for in vivo photodynamic and photothermal oral cancer therapies. Biomaterials.

[19] Yin W, Yan L, Yu J, Tian G, Zhou L, Zheng X, Zhang X, Yong Y, Li J, Gu Z, Zhao Y (2014). High-throughput synthesis of single-layer MoS_2_ nanosheets as a near-infrared photothermal-triggered drug delivery for effective cancer therapy. ACS Nano.

[20] Tao W, Ji X, Zhu X, Li L, Wang J, Zhang Y, Saw PE, Li W, Kong N, Islam MA (2018). Two-dimensional antimonene-based photonic nanomedicine for cancer theranostics. Adv Mater.

[21] Kong N, Zhang H, Feng C, Liu C, Xiao Y, Zhang X, Mei L, Kim JS, Tao W, Ji X (2021). Arsenene-mediated multiple independently targeted reactive oxygen species burst for cancer therapy. Nat Commun.

[22] Lee H, Dellatore SM, Miller WM, Messersmith PB (2007). Mussel-inspired surface chemistry for multifunctional coatings. Science.

[23] Yang J, Zhang X, Liu C, Wang Z, Deng L, Feng C, Tao W, Xu X, Cui W (2021). Biologically modified nanoparticles as theranostic bionanomaterials. Prog Mater Sci.

[24] Gao D, Chen T, Chen S, Ren X, Han Y, Li Y, Wang Y, Guo X, Wang H, Chen X (2021). Targeting hypoxic tumors with hybrid nanobullets for oxygen-independent synergistic photothermal and thermodynamic therapy. Nanomicro Lett.

[25] Ji X, Kang Y, Ouyang J, Chen Y, Artzi D, Zeng X, Xiao Y, Feng C, Qi B, Kim NY (2019). Synthesis of ultrathin biotite nanosheets as an intelligent theranostic platform for combination cancer therapy. Adv Sci.

[26] Yang K, Feng L, Shi X, Liu Z (2013). Nano-graphene in biomedicine: theranostic applications. Chem Soc Rev.

[27] Zhong X, Yang K, Dong Z, Yi X, Wang Y, Ge C, Zhao Y, Liu Z (2015). Polydopamine as a biocompatible multifunctional nanocarrier for combined radioisotope therapy and chemotherapy of cancer. Adv Func Mater.

[28] Liu X, Xie Z, Shi W, He Z, Liu Y, Su H, Sun Y, Ge D (2019). Polynorepinephrine nanoparticles: a novel photothermal nanoagent for chemo-photothermal cancer therapy. ACS Appl Mater Interfaces.

[29] Liu Y, Li Z, Yin Z, Zhang H, Gao Y, Huo G, Wu A, Zeng L (2020). Amplified photoacoustic signal and enhanced photothermal conversion of polydopamine-coated gold nanobipyramids for phototheranostics and synergistic chemotherapy. ACS Appl Mater Interfaces.

[30] Xue Y, Niu W, Wang M, Chen M, Guo Y, Lei B (2020). Engineering a biodegradable multifunctional antibacterial bioactive nanosystem for enhancing tumor photothermo-chemotherapy and bone regeneration. ACS Nano.

[31] She W, Luo K, Zhang C, Wang G, Geng Y, Li L, He B, Gu Z (2013). The potential of self-assembled, pH-responsive nanoparticles of mPEGylated peptide dendron-doxorubicin conjugates for cancer therapy. Biomaterials.

[32] Zhang X, Duan X, Hu Y, Tang Z, Miao C, Tao W, Wu J (2021). One-step and facile synthesis of peptide-like poly(melphalan) nanodrug for cancer therapy. Nano Today.

[33] Gao N, Nie J, Wang H, Xing C, Mei L, Xiong W, Zeng X, Peng Z (2018). A versatile platform based on black phosphorus nanosheets with enhanced stability for cancer synergistic therapy. J Biomed Nanotechnol.

[34] Cheng W, Nie J, Xu L, Liang C, Peng Y, Liu G, Wang T, Mei L, Huang L, Zeng X (2017). pH-sensitive delivery vehicle based on folic acid-conjugated polydopamine-modified mesoporous silica nanoparticles for targeted cancer therapy. ACS Appl Mater Interfaces.

[35] Zeng X, Luo M, Liu G, Wang X, Tao W, Lin Y, Ji X, Nie L, Mei L (2018). Polydopamine-modified black phosphorous nanocapsule with enhanced stability and photothermal performance for tumor multimodal treatments. Adv Sci.

[36] Lin H, Gao S, Dai C, Chen Y, Shi J (2017). A Two-dimensional biodegradable niobium carbide (MXene) for photothermal tumor eradication in NIR-I and NIR-II biowindows. J Am Chem Soc.

[37] Shvets P, Maksimova K, Demin M, Dikaya O, Goikhman A (2017). Cathodic arc sputtering of functional titanium oxide thin films, demonstrating resistive switching. Physica B.

[38] Frank O, Zukalova M, Laskova B, Kurti J, Koltai J, Kavan L (2012). Raman spectra of titanium dioxide (anatase, rutile) with identified oxygen isotopes (16, 17, 18). Phys Chem Chem Phys.

[39] Chen L, Wang D, Qiu J, Zhang X, Liu X, Qiao Y, Liu X (2021). Synergistic effects of immunoregulation and osteoinduction of ds-block elements on titanium surface. Bioact Mater.

[40] Wang L, Yu H, Wang K, Xu H, Wang S, Sun D (2016). Local fine structural insight into mechanism of electrochemical passivation of titanium. ACS Appl Mater Interfaces.

[41] Bertoti I, Mohai M, Sullivan J, Saied S (1995). Surface characterisation of plasma-nitrided titanium: an XPS study. Appl Surf Sci.

[42] Chakraborty S, Mukherjee J, Manna M, Ghosh P, Das S, Denys MB (2012). Effect of Ag nanoparticle addition and ultrasonic treatment on a stable TiO_2_ nanofluid. Ultrason Sonochem.

[43] Sakai N, Fujishima A, Watanabe T, Hashimoto K (2003). Quantitative evaluation of the photoinduced hydrophilic conversion properties of TiO_2_ thin film surfaces by the reciprocal of contact angle. J Phys Chem B.

[44] Chen H, Liu Z, Jiang O, Zhang J, Huang J, You X, Liang Z, Tao W, Wu J (2021). Nanocomposite of Au and black phosphorus quantum dots as versatile probes for amphibious SERS spectroscopy, 3D photoacoustic imaging and cancer therapy. Giant.

[45] Das G, Nicastri A, Coluccio ML, Gentile F, Candeloro P, Cojoc G, Liberale C, De Angelis F, Di Fabrizio E (2010). FT-IR, Raman, RRS measurements and DFT calculation for doxorubicin. Microsc Res Tech.

[46] Ji X, Ge L, Liu C, Tang Z, Xiao Y, Chen W, Lei Z, Gao W, Blake S, De D (2021). Capturing functional two-dimensional nanosheets from sandwich-structure vermiculite for cancer theranostics. Nat Commun.

[47] Habash RW, Bansal R, Krewski D, Alhafid HT (2006). Thermal therapy, part 1: an introduction to thermal therapy. Crit Rev Biomed Eng.

[48] Li Z, Hu Y, Howard KA, Jiang T, Fan X, Miao Z, Sun Y, Besenbacher F, Yu M (2016). Multifunctional bismuth selenide nanocomposites for antitumor thermo-chemotherapy and imaging. ACS Nano.

[49] Zeng X, Liu G, Tao W, Ma Y, Zhang X, He F, Pan J, Mei L, Pan G (2017). A drug-self-gated mesoporous antitumor nanoplatform based on pH-sensitive dynamic covalent bond. Adv Func Mater.

[50] Zhang A, Hai L, Wang T, Cheng H, Li M, He X, Wang K (2020). NIR-triggered drug delivery system based on phospholipid coated ordered mesoporous carbon for synergistic chemo-photothermal therapy of cancer cells. Chin Chem Lett.

[51] Gu M, Jiang L, Hao L, Lu J, Liu Z, Lei Z, Li Y, Hua C, Li W, Li X (2021). A novel theranostic nanoplatform for imaging-guided chemo-photothermal therapy in oral squamous cell carcinoma. J Mater Chem B.

[52] Qin Y, Guo Q, Wu S, Huang C, Zhang Z, Zhang L, Zhang L, Zhu D (2020). LHRH/TAT dual peptides-conjugated polymeric vesicles for PTT enhanced chemotherapy to overcome hepatocellular carcinoma. Chin Chem Lett.

[53] You X, Wang L, Wang L, Wu J (2021). Rebirth of aspirin synthesis by-product: prickly poly(salicylic acid) nanoparticles as self-anticancer drug carrier. Adv Funct Mater.

[54] Gao D, Guo X, Zhang X, Chen S, Wang Y, Chen T, Huang G, Gao Y, Tian Z, Yang Z (2020). Multifunctional phototheranostic nanomedicine for cancer imaging and treatment. Mater Today Bio.

[55] Pan C, Ou M, Cheng Q, Zhou Y, Yu Y, Li Z, Zhang F, Xia D, Mei L, Ji X (2020). Z-Scheme heterojunction functionalized pyrite nanosheets for modulating tumor microenvironment and strengthening photo/chemodynamic therapeutic effects. Adv Func Mater.

[56] Zhang Z, Wang J, Chen C (2013). Near-infrared light-mediated nanoplatforms for cancer thermo-chemotherapy and optical imaging. Adv Mater.

