# Supplementary material for: Titanium nanosheet as robust and biosafe drug carrier for combined photochemo cancer therapy
Source: J Nanobiotechnology. 2022 Mar 24;20:154. doi: 10.1186/s12951-022-01374-0 (PMC8944145; doi:10.1186/s12951-022-01374-0)
Supplement: Supplementary file 1 — Additional file 1: Figure S1. DOX loading efficiencies on Ti@PDA-PEG NSs (%) with increasing DOX feeding concentrations. Figure S2. (A, B) TEM images of Ti@PDA-PEG NSs, and (C, D) TEM images of Ti@PDA-PEG-DOX NSs. Figure S3. DLS size distribution of different samples. Figure S4. UV–vis–NIR absorption spectra of Ti and Ti@PDA-PEG-DOX NSs. Figure S5. Photo images of the Ti@PDA-PEG-DOX NSs incubated in water, PBS and cell culture. Figure S6. Stability of Ti@PDA-PEG-DOX NSs in water during 72 h. Figure S7. Histology analysis of the major organs extracted from tumor bearing mice stained with hematoxylin and eosin (H&E) after treatment with saline, Ti@PDA-PEG NSs + NIR, free DOX,Ti@PDA-PEG-DOX NSs, Ti@PDA-PEG-DOX NSs + NIR for 14 days (scale bar = 50 μm). [file 12951_2022_1374_MOESM1_ESM.docx]

**Titanium Nanosheet as Robust and Biosafe Drug Carrier for Combined Photochemo Cancer Therapy**

Xiaoli Yuan ^‡ 1^, Ying Zhu ^‡ 1^, Shasha Li^3^, Yiqun Wu^1^, Zhongshi Wang^1^, Rui Gao^1^, Shiyao Luo^1^, Juan Shen^1^,Jun Wu^2*^ and Liang Ge^1*^

^*^Correspondence: wujun29@mail.sysu.edu.cn; geliang1981@hotmail.com

^1^ State Key Laboratory of Natural Medicines, China Pharmaceutical University, No.24 Tongjia Xiang, Nanjing, 210009, China

^2^ School of Biomedical Engineering, Sun Yat-sen University, Guangzhou 510006, China

^3^School of Pharmacy, Xinjiang Medical University, Xinjiang 830000, China

‡ The authors equally contributed to this work.


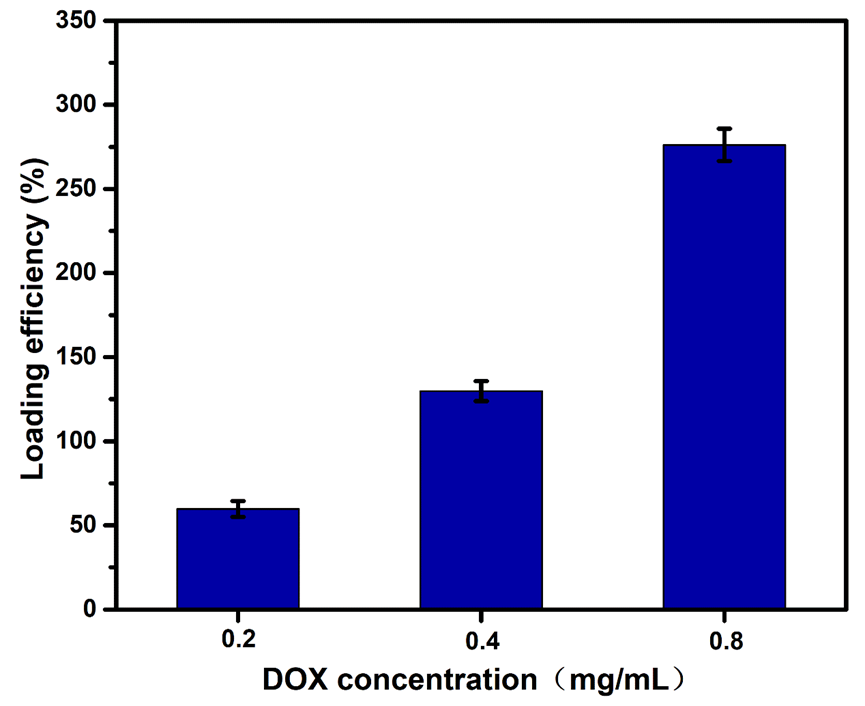


Figure S1. DOX loading efficiencies on Ti@PDA-PEG NSs (%) with increasing DOX feeding concentrations.





Figure S2. (A, B) TEM images of Ti@PDA-PEG NSs, and (C, D) TEM images of Ti@PDA-PEG-DOX NSs


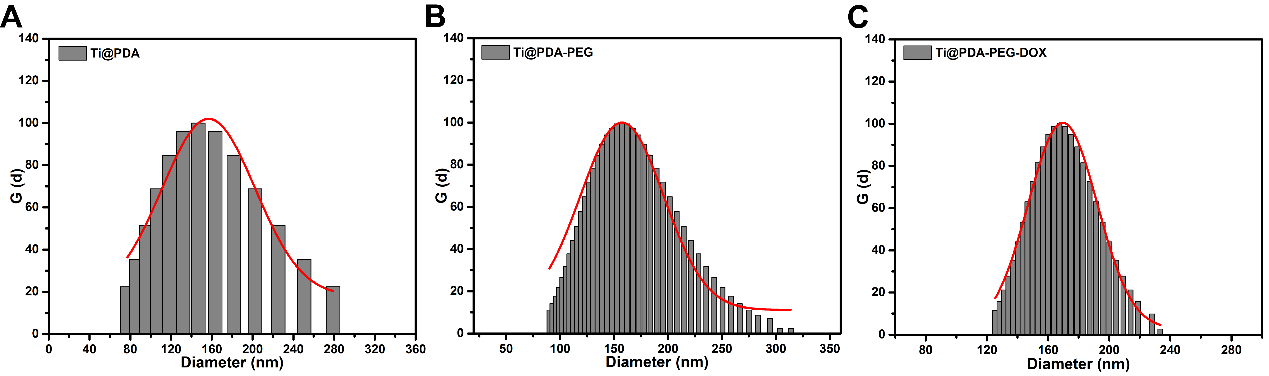


Figure S3. DLS size distribution of different samples.





Figure S4. UV−vis-NIR absorption spectra of Ti and Ti@PDA-PEG-DOX NSs.


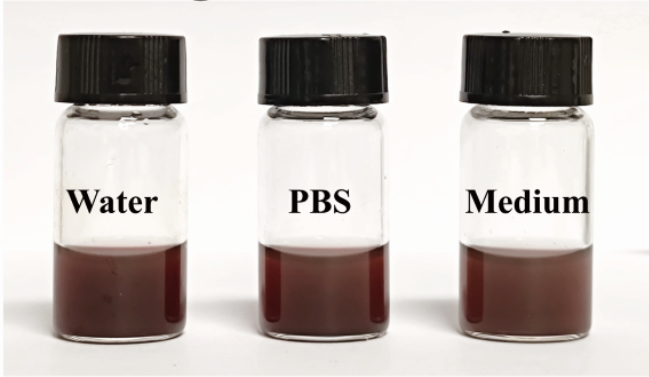


Figure S5. Photo images of the Ti@PDA-PEG-DOX NSs incubated in water, PBS and cell culture medium after 72 h.





Figure S6. Stability of Ti@PDA-PEG-DOX NSs in water during 72h.





Figure S7. Histology analysis of the major organs extracted from tumor bearing mice stained with hematoxylin and eosin (H&E) after treatment with saline, Ti@PDA-PEG NSs+NIR, free DOX,Ti@PDA-PEG-DOX NSs, Ti@PDA-PEG-DOX NSs+NIR for 14 days. (scale bar = 50μm)
